# Descriptive analysis about the use of ultrasound in fourth-year medical students at University of Lleida (UdL)

**DOI:** 10.1186/2036-7902-6-S1-A9

**Published:** 2014-01-31

**Authors:** Ramon Nogué Bou, Nuria Escudero García, N Anna Habimana Jordana, José Javier Trujillano Cabello, Jesús Pérez Mur, Anna Nogué Infante

**Affiliations:** 1 University of Lleida Spain

## Objective

To observe if a sample of 4^th^-year medical students of Emergency Medicine at the University of Medicine of Udl, can acquire theoretical and practical knowledge of ultrasound and assess whether that knowledge increases the perceived significance of this diagnostic test for their future medical practice. There is not any evidence of previous publications about the matter in our country.

## Methods

The students completed a questionnaire before and after a 2-hour practical lesson about ultrasound. The surveys were divided in three parts: a) questions about theoretical and practical knowledge about ultrasound using a 5-point Likert scale of perceived importance ; b) list of structures and pathologies that the students were able to detect by using the ultrasound; c) open-ended questions and comments. The statisitics are presented as percent means or proportions with p-values from t-testing.

## Results

Significant improvements in knowledge about ultrasound were observed, comparing the pre- and post-practical tests (the number of structures and pathologies detected increased). The intervention also increased their perceived importance of this diagnostic tool.

## Conclusion

The students increased their level of knowledge about ultrasound, as well as the level of the perceived importance of its use in medical practice. It would be interesting to conduct more educational sessions on this diagnostic tool, including the introduction of ultrasound in different subjects, including anatomy and physiology during the pre-clinical years of training, in our country as it is done in other countries.

